# Improving risk assessment in forensic mental health: Temporal validation and clinical refinement of the FoVOx risk tool

**DOI:** 10.1192/j.eurpsy.2026.12223

**Published:** 2026-05-25

**Authors:** Lenka Sivak, Jonas Forsman, Amir Sariaslan, Jari Tiihonen, Seena Fazel

**Affiliations:** 1 Department of Clinical Neuroscience, Karolinska Institutet, Sweden; 2 National Board of Forensic Medicine, Sweden; 3 Department of Psychiatry, University of Oxford, UK and Oxford Health NHS Foundation Trust; 4 Department of Forensic Psychiatry, University of Eastern Finland, Finland

**Keywords:** forensic psychiatry, risk assessment, prediction model, violence

## Abstract

**Background:**

Forensic psychiatric services are expanding in many countries, and discharging patients from secure hospitals relies on accurate estimates of risk of adverse outcomes. Novel evidence-based tools for estimating one key risk, violent reoffending, have been developed in recent years. We aimed to externally validate one new tool, FoVOx, in forensic psychiatric patients sentenced to treatment and to develop an updated model (FoVOx2), incorporating additional clinical predictors.

**Methods:**

Using Swedish national registers, we conducted a temporal external validation of FoVOx by examining 767 patients discharged between 2014 and 2023. For the FoVOx2 cohort, 906 patients discharged between 2008 and 2023 were followed up, and additional predictors tested. The outcome was violent reconviction within 12 or 24 months. Model performance was evaluated using Harrell’s C-index, area under the receiver operating characteristics curves (AUCs), calibration, and classification metrics at predefined thresholds.

**Results:**

In temporal validation, FoVOx showed moderate discrimination (AUCs 0.69 and 0.71; C-index = 0.69) and acceptable overall accuracy (Brier <0.11). Calibration was generally good, with mild overestimation at the highest predicted risks (>20%) at 12 months and slight underprediction at 24 months. The updated FoVOx2 model incorporated novel predictors, including clozapine treatment and additional diagnostic categories. It was associated with improved performance (AUCs 0.77; optimism-corrected C-index = 0.72; Brier 0.06 and 0.09) and demonstrated good calibration (intercept ≈ 0; slopes 1.03 and 1.05).

**Conclusions:**

Updating risk assessment tools with additional clinical factors can lead to incremental improvement in model performance. Implementing tools should consider clinical utility and impact as next steps.

## Introduction

Preventing violent recidivism (or repeat violent offending) after discharge from forensic psychiatric care is a key priority for patients and their carers, clinical services, and wider society. Rates of violent reoffending range from 3,000 to 4,500 offences per 100,000 person-years, which have been reported in national cohort studies [1–3]. Decisions about discharge from forensic services are based on many clinical and legal considerations, with a key one related to reoffending risks [4]. These decisions carry significant implications for resource allocation for community mental health services, and more widely for public health and safety, as well as potential reduction of disproportionate detention, making accurate and transparent risk assessments an integral part of high-quality risk management and safe services [1, 5–12].

Traditional approaches relying on unstructured clinical judgment have shown limited reliability and predictive validity [13, 14]. In contrast, structured methods – professional judgment (SPJ) instruments and probabilistic (actuarial) models – provide more accurate and transparent risk estimates [15–17]. In particular actuarial tools, developed using empirically-derived risk markers, offer standardized, time-efficient, and consistent assessments with quantifiable risk estimates [18, 19]. They are especially useful in forensic psychiatry, where clear risk communication across judicial, social, and healthcare systems is necessary [20].

FoVOx was developed using Swedish data as a probabilistic risk tool for predicting violent reoffending following discharge from forensic psychiatric care [5]. The predictors for the tool comprise routinely collected sociodemographic and clinical data, and the risk model is intended to complement, rather than replace, clinical evaluations by providing a baseline level of risk [21]. The internally validated FoVOx model demonstrated good discriminatory accuracy (area under the receiver operating characteristics curve [AUC] = 0.77) in a nationwide cohort and translated into a 12-item online risk tool that is easy to implement and does not require specific training for use by clinicians. Thus, the tool is highly applicable and optimized for clinical settings where time and resources are limited [5, 22–24].

However, the original cohort is now more than a decade old, and included a heterogeneous sample of people legally sentenced to forensic psychiatric care and those treated by forensic psychiatric services in prison. The former population is mandated to undergo regular psychiatric monitoring and faces legally sanctioned restrictions on discharge, which will alter reoffending risk [25, 26] – and is broadly comparable to patients in forensic psychiatric hospitals in most high-income countries (where people are sent to forensic hospitals under various legal sanctions, such as the Mental Health Act in England and Wales). Comparative European data suggest similar patient profiles (predominantly male, high prevalence of psychotic disorders and comorbid substance use) although there is variation in admission thresholds, length of stay, bed availability, and use of compulsory outpatient care [8, 27, 28].

Therefore, this study had two aims. The first aim was to conduct a temporal external validation of FoVOx among sentenced forensic psychiatric patients discharged between 2014 and 2023. The second was to update and internally validate the model (FoVOx2) using a more recent cohort of sentenced patients discharged between 2008 and 2023.

## Material and methods

### Data sources and linkage

Registers were cross-linked using Sweden’s unique 12-digit personal identity number, issued by the Swedish Tax Agency and used reliably across administrative and health registers [29]. The following databases were used:

- National Board of Forensic Medicine’s records: An internal database, covering all forensic psychiatric evaluations conducted in Sweden, available since 2009 [30].
- National Forensic Psychiatric Register (NFPR): A national quality registry, established in 2008, that monitors both inpatient and outpatient forensic psychiatric care, including diagnoses, treatments, and medication. In 2023, coverage included 96% of units and 84% of sentenced patients [26].
- National Patient Register (NPR): covering all inpatient admissions nationwide since 1987 [31].
- Longitudinal Integration Database for Health Insurance and Labour Market Studies (LISA): A comprehensive database containing annual socioeconomic information (e.g., age, sex, employment, education, income, benefits) since 1990.
- National Council for Crime Prevention’s Crime Register: Official data for convictions, including offence type, date of offence, and sentencing details [32].
- Cause of Death Register: A complete national register of all deaths since 1952.

### Study populations

The temporal validation cohort included individuals who underwent forensic psychiatric evaluation according to the National Board of Forensic Medicine’s records (since January 2009), were sentenced to forensic psychiatric care and later discharged between January 2014 and December 2023 (n = 767) according to NFPR. In Sweden, forensic psychiatric care is imposed by a criminal court after an offence when the individual is assessed to have had a severe mental disorder at the time, typically based on a forensic psychiatric evaluation. It is not limited to serious violent offences; the key criterion is the presence of a severe mental disorder in relation to the offence. Courts may also order special court supervision (“särskild utskrivningsprövning”) for individuals considered at higher risk, requiring stricter judicial oversight. Unlike prison sentences, forensic psychiatric care is not time-limited. Instead, it is subject to regular judicial review, with discharge determined by the courts based on ongoing risk, clinical status, and treatment progress. NFPR records both inpatient and outpatient compulsory care; hence, “discharge” in this study denotes total discharge from forensic services (i.e., after outpatient part of their forensic care has also ended). This measure was chosen for both practical and clinical reasons: many patients transition repeatedly between inpatient and outpatient settings, and both forms of care constitute compulsory treatment with a high level of supervision, oversight, and control [25, 26]. Also, this definition reflects the point at which individuals are no longer subject to legally mandated forensic restrictions and are living in the community, with time at risk to be arrested, charged, and sentenced for new community-based offences. From a risk assessment perspective, this represents the clinically relevant time point for estimating violent reoffending. It is important to note that discharge pathways and legal frameworks differ across jurisdictions. In some systems, such as England, many patients transition from secure forensic inpatient care to general adult psychiatric services without ongoing forensic legal oversight, whereas a smaller subgroup remains under continued forensic supervision (restriction orders) even while living in the community. Consequently, the level of supervision and legal constraint following discharge can vary. The definition of discharge used in this study is therefore most directly comparable to transitions into community living without active forensic restrictions. While this supports some generalisability of the outcome definition, differences in post-discharge legal status and supervision should be considered when considering the model in other national settings. For individuals with multiple treatment periods, one episode was randomly selected to avoid bias due to repeated observations. The updated cohort to develop a new model comprised sentenced patients totally discharged between November 2008 and December 2023 (n = 906). This larger cohort partially overlapped with the validation sample but extended the timeframe to include earlier discharges and augment statistical power.

### Outcome

The primary outcome was conviction for a violent crime committed after total discharge within 12 and 24 months, irrespective of when the conviction occurred. Violent crimes included homicide, assault, robbery, arson, sexual offences, and unlawful threats or harassment [33]. The date of the offence was used as the event time to minimise bias from judicial processing delays.

### Predictors

Sociodemographic data, including age, sex, and employment status before admission, were obtained from LISA. Information on previous convictions of violent and serious violent crimes was extracted from the Crime Register. Serious violent crime was defined as homicide or manslaughter, aggravated assault, aggravated robbery, aggravated arson, rape, sexual coercion, or sexual exploitation. The NPR was used to identify the number of previous psychiatric inpatient treatments. The NFPR provided data on psychiatric and substance use diagnoses, psychotropic medication, and treatment duration. Information on medication at discharge was classified into the following groups: oral antipsychotics (except clozapine); clozapine; long-acting injectable (LAI) antipsychotics; mood stabilizers/antiepileptics (including lithium); and antidepressants. Diagnoses were classified to main categories using ICD-10: schizophrenia-spectrum disorders (F20–F29), bipolar disorder (F30–F31), unipolar depression (F32–F34.1), anxiety and stress-related disorders (F41, F43–F45, F48), antisocial personality disorder (F60.2), other personality disorders (F60–F62 excl. F60.2, F69), attention-deficit/hyperactivity disorder (ADHD) (F90), developmental disorders (F70–F79, F84), alcohol use disorder (F10), and drug use disorder (F11–F19). In the temporal validation, the original FoVOx model structure was maintained, in which a single primary diagnosis was used per patient. The new FoVOx2 model allowed for overlapping diagnoses to better reflect clinical practice where psychiatric comorbidities are common, and the assignment of a “primary” diagnosis can be inconsistent [34, 35]. In addition, for the updated model, we dropped two predictors: employment before admission and treatment duration shorter than 1 year as they were not statistically significant in preliminary analyses. The variable for number of previous inpatient episodes was simplified to a binary indicator (yes/no) to improve clinical feasibility and ease of implementation [23, 24].

### Model development and validation

For the temporal validation, the original FoVOx coefficients were applied to compute individual linear predictors and predicted risks of violent reoffending at 12 and 24 months after total discharge from compulsory forensic psychiatric care [5]. For model validation, missing data were handled using multiple imputation by chained equations [36]. Data were censored for outcome, mortality, emigration, or end of follow-up.

For the updated FoVOx2 model, the original FoVOx analytic protocol for predictor selection was used, employing a two-stage Cox proportional hazards approach. In the first stage, one set of predictors – sex, age at discharge, previous violent crime, schizophrenia-spectrum disorder, alcohol use disorder, and other substance use disorder – were included based on prior evidence and clinical relevance [3, 34, 37–39]. In the second stage, additional predictors were entered by stepwise selection until no variables remained with a p-value greater than 0.1 in multivariable models (aligning with original model development).

### Further statistical analyses

Discrimination was evaluated using two complementary metrics. The Harrell’s C-index measured the model’s overall ability to rank individuals by risk across the entire follow-up period (global concordance), inherently accounting for censoring. In addition, time-dependent AUCs were calculated at 12 and 24 months to assess discrimination at specific time horizons, using inverse probability of censoring weighting (IPCW) to adjust for right-censoring. IPCW is a weighting approach in which each individual’s contribution is scaled by the inverse of the estimated probability of not being censored at a given time, thus mitigating the right-censoring bias [40]. Calibration was assessed through calibration slopes and calibration plots comparing observed and predicted risks [41]. Overall calibration accuracy was estimated using IPCW-adjusted Brier scores [42].

Classification performance was summarised by sensitivity, specificity, positive predictive value (PPV), and negative predictive value (NPV) at prespecified risk thresholds of 5% and 20%, in line with the original FoVOx reporting. The proportional hazards assumption was tested using scaled Schoenfeld residuals. Internal validation of the new models was performed using bootstrap resampling (500 samples) to estimate and correct for optimism in performance metrics (C-index, calibration slope, Dxy, and R^2^). [43] Adequacy of our sample size was ensured by maintaining approximately 10 events of reoffending per candidate predictor – a recommended guideline to reduce the risk of overfitting in prediction modelling [44, 45].

All analyses were conducted using R version 4.4.2. The date of offence was used as the event time, and incomplete follow-up was handled by censoring, consistent with standard survival analysis methods [46]. In addition to the main analyses, tables, and figures presented in this article, additional material – including a psychosis-specific model – is provided in the supplementary material.

### Ethics approval

Ethical approval for this study was obtained from the Swedish Ethical Review Authority (case number 2023-04161-01). All data were pseudonymised prior to analysis. Inclusion to the NFPR was based on an informed consent obtained at the time of first registration.

## Results

### Cohort characteristics

The temporal validation cohort comprised 767 individuals sentenced to forensic psychiatric care and fully discharged between January 2014 and December 2023. During follow-up, 118 patients (15.4%) were convicted of a new violent offence. The mean age at discharge was 42 years, and 79% were male. More than half (54.2%) were diagnosed with schizophrenia-spectrum disorder, and approximately half (44.9%) had some form of substance use disorder. Median treatment duration was 54 months. The FoVOx2 development cohort included 906 sentenced patients discharged between November 2008 and December 2023, of whom 172 (19.0%) reoffended violently. Baseline characteristics for both cohorts are summarised in Tables 1 and 2.

**Table 1.** Temporal validation cohort. Individuals sentenced to forensic psychiatric treatment in Sweden, discharged between January 2014 and December 2023 Table 1. long description.

	**No violent recidivism**	**Violent recidivism**	**Overall**
Number of observations	649	118	767
**Time to relapse/censoring**			
Mean, months (SD)	47.9 (33.3)	22.1 (20.6)	43.9 (33.0)
Median, months [min, max]	41.5 [0.131, 119]	16.0 [0.296, 93.4]	36.1 [0.131, 119]
**Sex**			
Male, n	502 (77.3%)	104 (88.1%)	606 (79.0%)
Female, n	147 (22.7%)	14 (11.9%)	161 (21.0%)
**Age at discharge**			
Mean, years (SD)	43.5 (13.7)	36.2 (9.75)	42.4 (13.4)
Median, years [min, max]	41.0 [20.0, 92.0]	33.5 [22.0, 65.0]	39.0 [20.0, 92.0]
**Duration of forensic psychiatric treatment**			
Mean, months (SD)	60.3 (34.0)	57.4 (39.9)	59.9 (35.0)
Median, months [min, max]	55.2 [3.88, 227]	46.0 [3.75, 213]	54.0 [3.75, 227]
**Personality disorder at discharge**			
No, n	552 (85.1%)	89 (75.4%)	641 (83.6%)
Yes, n	97 (14.9%)	29 (24.6%)	126 (16.4%)
**Previous violent crime**			
No, n	75 (11.6%)	4 (3.4%)	79 (10.3%)
Yes, n	574 (88.4%)	113 (96.6%)	688 (89.7%)
**Previous serious violent crime**			
No	477 (73.5%)	84 (71.2%)	561 (73.1%)
Yes	172 (26.5%)	34 (28.8%)	206 (26.9%)
**Primary diagnosis at discharge**			
Schizophrenia spectrum disorder, n	355 (54.7%)	61 (51.7%)	416 (54.2%)
Bipolar disorder, n	48 (7.4%)	11 (9.3%)	59 (7.7%)
Unipolar depression, n	12 (1.8%)	0 (0%)	12 (1.6%)
Anxiety disorder, n	9 (1.4%)	0 (0%)	9 (1.2%)
Other, n	225 (34.7%)	46 (39.0%)	271 (35.3%)
**Drug use disorder at discharge**			
No, n	449 (69.2%)	49 (41.5%)	498 (64.9%)
Yes, n	200 (30.8%)	69 (58.5%)	269 (35.1%)
**Lifetime drug use disorder**			
No, n	279 (43.0%)	28 (23.7%)	307 (40.0%)
Yes, n	350 (53.9%)	89 (75.4%)	439 (57.2%)
Missing, n	20 (3.1%)	1 (0.8%)	21 (2.7%)
**Alcohol use disorder at discharge**			
No, n	552 (85.1%)	100 (84.7%)	652 (85.0%)
Yes, n	97 (14.9%)	18 (15.3%)	115 (15.0%)
**Employment before admission**			
No, n	555 (85.5%)	106 (89.8%)	661 (86.2%)
Yes, n	88 (13.6%)	10 (8.5%)	98 (12.8%)
Missing, n	6 (0.9%)	2 (1.7%)	8 (1.0%)
**Number of previous inpatient episodes** **(five or more)**			
No, n	370 (57.0%)	54 (45.8%)	424 (55.3%)
Yes, n	279 (43.0%)	64 (54.2%)	343 (44.7%)
**Duration of forensic psychiatric treatment** **(12 months or more)**			
No, n	26 (4.0%)	9 (7.6%)	35 (4.6%)
Yes, n	623 (96.0%)	109 (92.4%)	732 (95.4%)

**Table 2.** Updated cohort. Individuals sentenced to forensic psychiatric treatment in Sweden, discharged between November 2008 and December 2023 Table 2. long description.

	**No violent recidivism**	**Violent recidivism**	**Overall**
Number of observations	734	172	906
**Time to relapse/censoring**			
Mean, months (SD)	55.4 (40.5)	28.5 (26.7)	51.6 (40.1)
Median, months [min, max]	47.6 [0.131, 173]	20.3 [0.296, 132]	39.8 [0.131, 173]
**Sex**			
Male, n	572 (77.9%)	148 (86.0%)	720 (79.5%)
Female, n	162 (22.1%)	24 (14.0%)	186 (20.5%)
**Age at discharge**			
Mean, years (SD)	43.6 (13.7)	37.4 (10.5)	42.4 (13.4)
Median, years [min, max]	41.0 [18.0, 92.0]	35.0 [20.0, 68.0]	40.0 [18.0, 92.0]
**Duration of forensic psychiatric treatment**			
Mean, months (SD)	56.1 (34.1)	54.9 (39.1)	55.9 (35.1)
Median, months [min, max]	50.4 [2.17, 227]	45.5 [3.75, 213]	48.9 [2.17, 227]
**Previous violent crime**			
No, n	86 (11.7%)	7 (4.1%)	93 (10.3%)
Yes, n	648 (88.3%)	165 (95.9%)	813 (89.7%)
**Previous serious violent crime**			
No	541 (73.7%)	129 (75.0%)	670 (74.0%)
Yes	193 (26.3%)	43 (25.0%)	236 (26.0%)
**Schizophrenia spectrum disorder**			
No, n	212 (28.9%)	48 (27.9%)	260 (28.7%)
Yes, n	522 (71.1%)	124 (72.1%)	646 (71.3%)
**Affective disorder**			
No, n	601 (81.9%)	145 (84.3%)	746 (82.3%)
Yes, n	133 (18.1%)	27 (15.7%)	160 (17.7%)
**Developmental disorder (ID/ASD)**			
No, n	425 (57.9%)	78 (45.3%)	503 (55.5%)
Yes, n	309 (42.1%)	94 (54.7%)	403 (44.5%)
**ADHD**			
No, n	652 (88.8%)	143 (83.1%)	795 (87.7%)
Yes, n	82 (11.2%)	29 (16.9%)	111 (12.3%)
**Antisocial personality disorder**			
No, n	701 (95.5%)	145 (84.3%)	846 (93.4%)
Yes, n	33 (4.5%)	27 (15.7%)	60 (6.6%)
**Personality disorder, other than antisocial**			
No, n	629 (85.7%)	137 (79.7%)	766 (84.5%)
Yes, n	105 (14.3%)	35 (20.3%)	140 (15.5%)
**Drug use disorder**			
No, n	521 (71.0%)	77 (44.8%)	598 (66.0%)
Yes, n	213 (29.0%)	95 (55.2%)	308 (34.0%)
**Alcohol use disorder**			
No, n	627 (85.4%)	145 (84.3%)	772 (85.2%)
Yes, n	107 (14.6%)	27 (15.7%)	134 (14.8%)
**Previous psychiatric inpatient treatment**			
No, n	96 (13.1%)	11 (6.4%)	107 (11.8%)
Yes, n	638 (86.9%)	161 (93.6%)	799 (88.2%)
**Long-acting injectables**			
No, n	484 (65.9%)	122 (70.9%)	606 (66.9%)
Yes, n	250 (34.1%)	50 (29.1%)	300 (33.1%)
**Oral antipsychotics**			
No, n	521 (71.0%)	122 (70.9%)	643 (71.0%)
Yes, n	213 (29.0%)	50 (29.1%)	263 (29.0%)
**Clozapine**			
No, n	645 (87.9%)	166 (96.5%)	811 (89.5%)
Yes, n	89 (12.1%)	6 (3.5%)	95 (10.5%)
**Mood stabilizers**			
No, n	612 (83.4%)	149 (86.6%)	761 (84.0%)
Yes, n	122 (16.6%)	23 (13.4%)	145 (16.0%)
**Antidepressants**			
No, n	515 (70.2%)	138 (80.2%)	653 (72.1%)
Yes, n	219 (29.8%)	34 (19.8%)	253 (27.9%)

### Temporal validation of FoVOx

**Figure 1.**
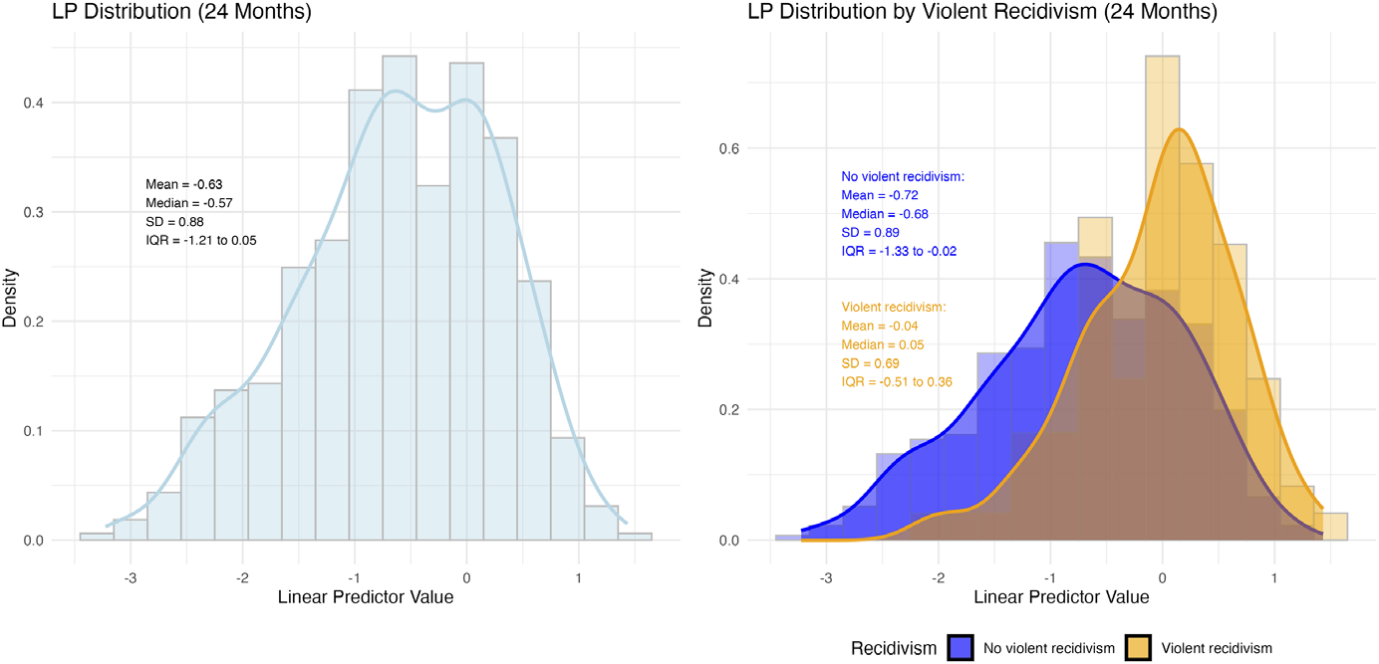
Distributions of the model’s linear predictor (LP) values for individuals with and without violent recidivism 24 months. Figure 1. long description.

**Figure 2.**
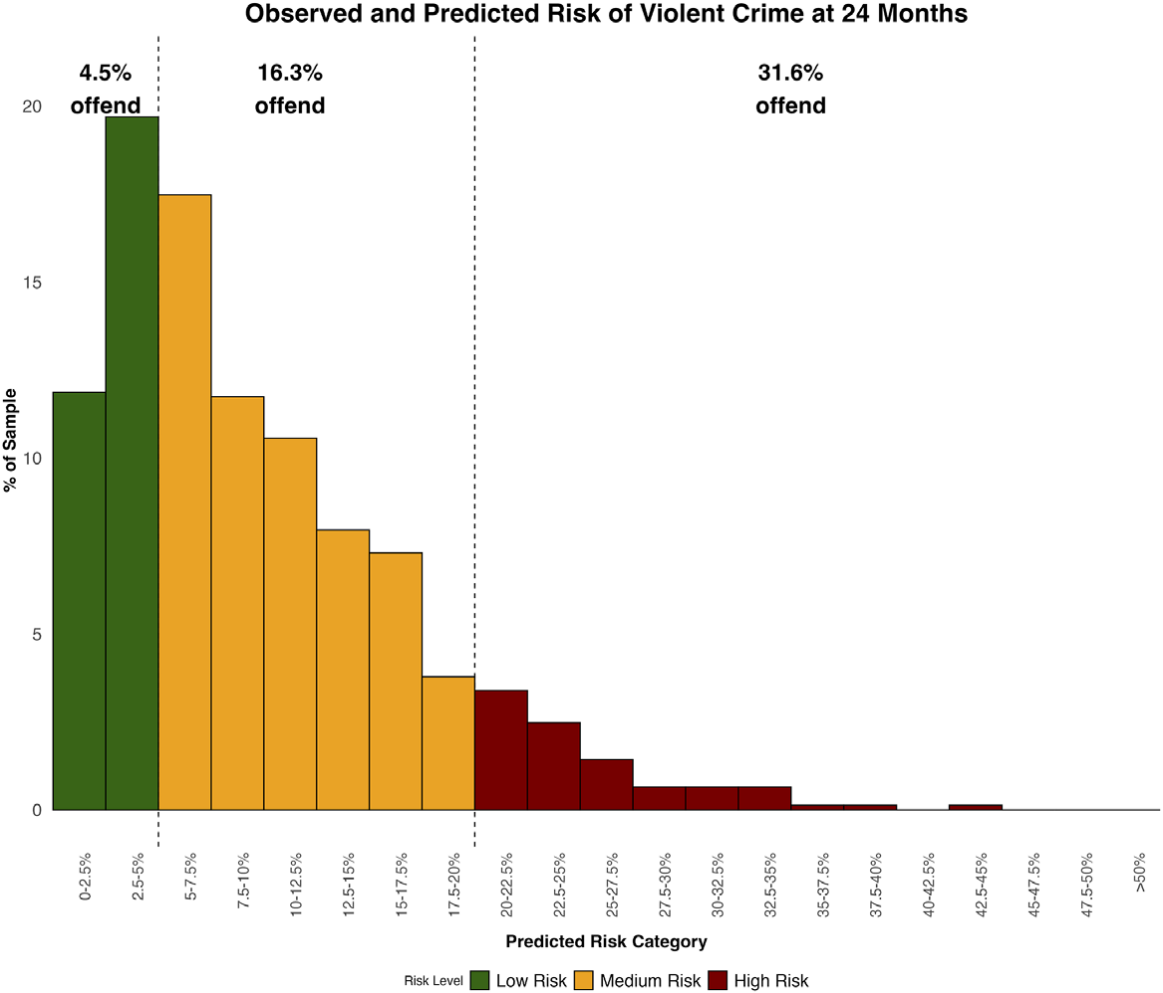
Observed and predicted risk of violent crime at 24 months, by risk category. Figure 2. long description.

In the sentenced forensic cohort, the original FoVOx model demonstrated moderately good discriminatory accuracy across the full follow-up period (C-index 0.69 [95% CI 0.64–0.73], as well as at fixed time horizons, with AUCs of 0.69 at 12 months and 0.71 at 24 months (Appendix Figure 1). Figure 1 and Appendix Figure 2 present the distribution of the linear predictor (LP) values by violent recidivism status. The distribution of predicted risks at fixed time points was concentrated toward the lower end, with most individuals below 10% and few exceeding 20% (Figure 2; Appendix Figure 3). Accordingly, because most individuals were classified into lower risk categories, a substantial proportion of violent outcomes arose from these groups despite lower individual risk, reflecting the underlying distribution of risk rather than misclassification.

**Figure 3.**
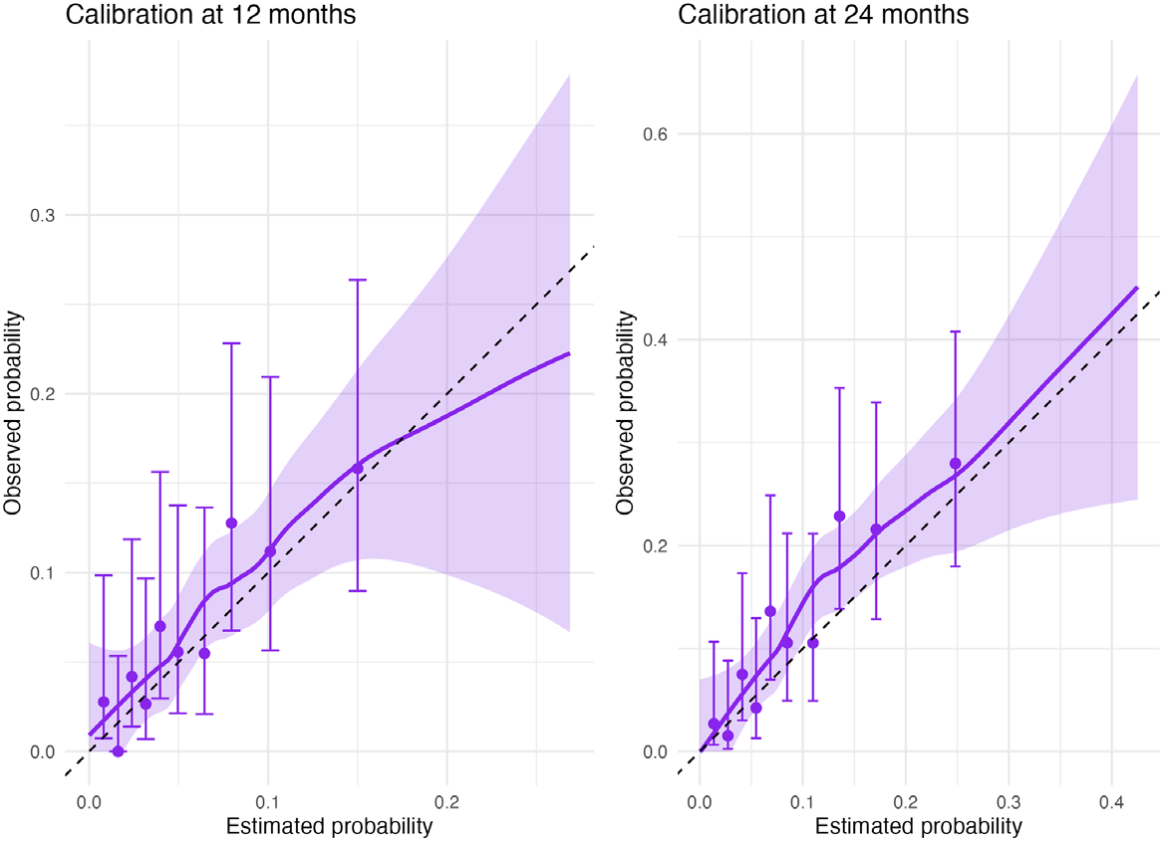
Calibration plots for FoVOx temporal validation. Figure 3. long description.

The model displayed well-calibrated risk estimates overall. At 12 months, the model showed mild overprediction and some imprecision (intercept −0.26; calibration slope 0.83). At 24 months, calibration was good, with an intercept of 0.20 and a slope of 0.95, indicating slight underprediction. Brier scores of 0.061 and 0.101 supported acceptable overall accuracy. At a 5% threshold, sensitivity/specificity were 0.70/0.57 (12 months) and 0.90/0.35 (24 months); at 20%, the trade-off reversed (0.02/0.99 and 0.22/0.93, respectively). Calibration plots (Figure 3) showed good alignment at lower predicted probabilities; at 12 months there was modest overestimation in the upper range, whereas at 24 months, the model consistently underpredicted risk.

### FoVOx2: Updated model and internal validation

In the new development cohort (n = 906, violent offences [events] = 172), the regression analyses identified additional independent predictors: antisocial personality disorder, other personality disorder, previous psychiatric inpatient treatment, and clozapine treatment. Older age, female sex, absence of previous inpatient episodes, and clozapine at discharge were associated with lower risk, whereas antisocial personality disorder, other types of personality disorders, drug use disorders, and previous violence markedly increased risk. Hazard ratios ranged from 0.3 (95% CI 0.1–0.6) for clozapine treatment to 3.9 (95% CI 1.8–8.3) for a history of violent offending (Table 3).

**Table 3.** Association between predictors and violent reoffending in the updated model (FoVOx2), derived using a two-stage Cox proportional hazards regression Table 3. long description.

**Predictor**	**HR**	**95% CI**	**p-value**
Sex (female)	0.61	0.39–0.95	0.029
Age at discharge (per 1-yr older)	0.97	0.95–0.98	<0.001
Previous violent crime	3.90	1.82–8.35	<0.001
Schizophrenia spectrum disorder	1.19	0.83–1.69	0.348
Alcohol use disorder	1.25	0.82–1.91	0.309
Substance use disorder, other than alcohol	2.34	1.70–3.22	<0.001
Antisocial personality disorder	3.37	2.18–5.23	<0.001
Personality disorder, other than antisocial	1.70	1.13–2.54	0.011
Clozapine treatment	0.28	0.12–0.63	0.002
Absence of previous psychiatric inpatient care	0.58	0.31–1.07	0.082

**Figure 4.**
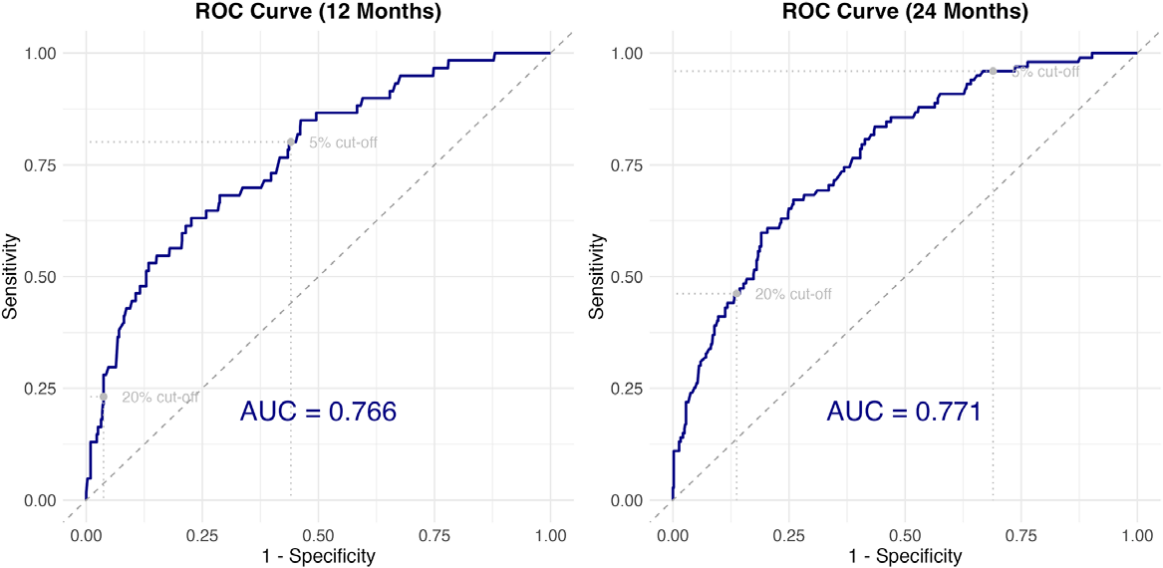
FoVOx2 model discrimination, presented as receiver operating characteristics (ROC) curves. Figure 4. long description.

**Figure 5.**
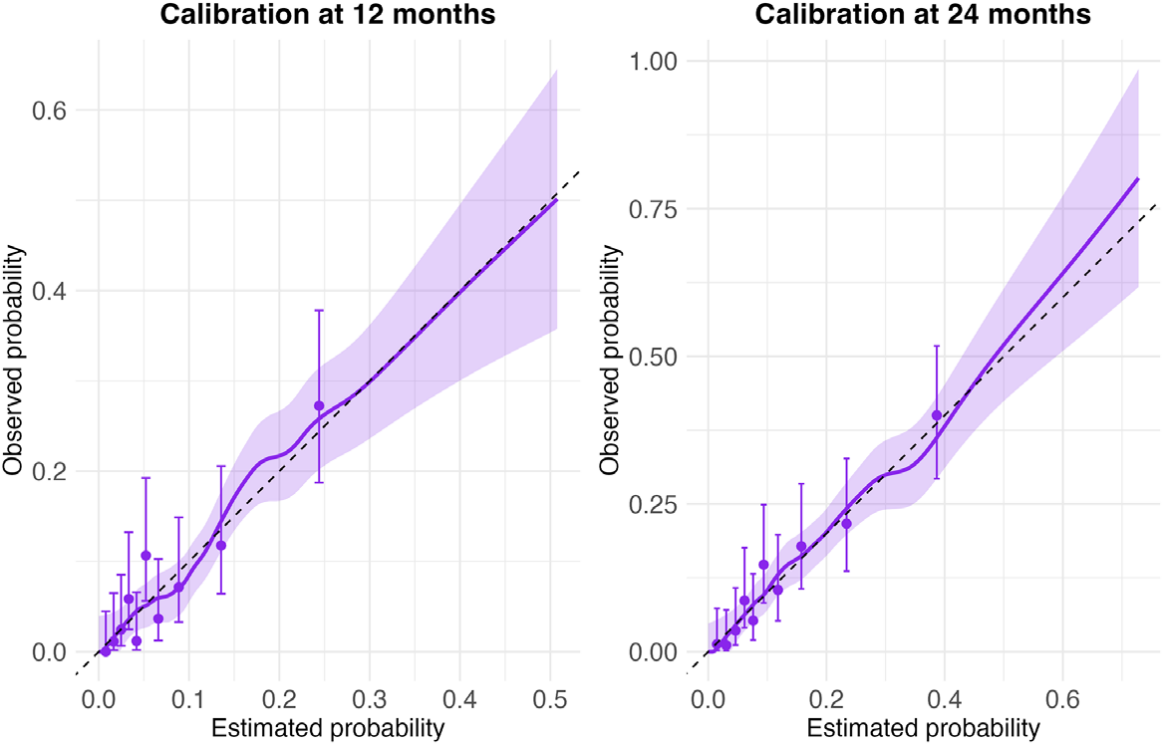
Calibration plots for FoVOx2 at 12 and 24 months. Figure 5. long description.

**Figure 6.**
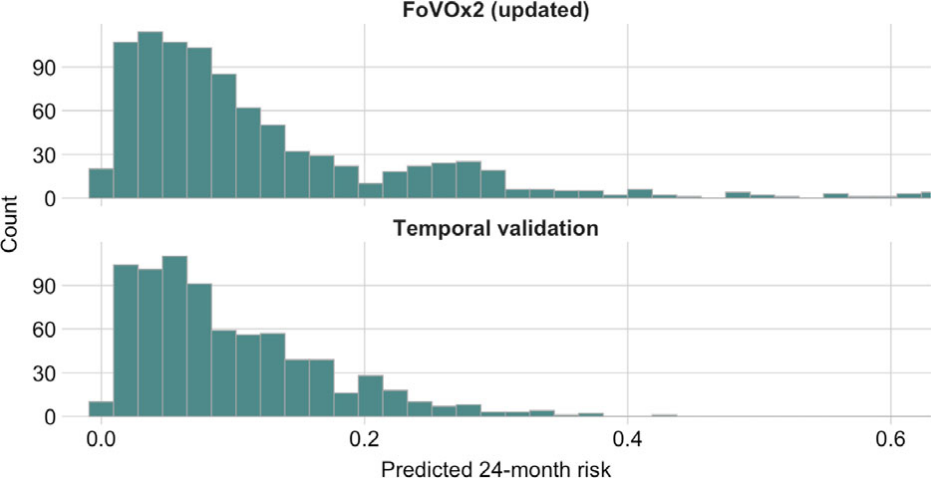
Predicted 24-month risk distributions: FoVOx2 (updated) vs. temporal validation. Figure 6. long description.

The model showed an apparent C-index of 0.75 (95% CI 0.71–0.78) and an optimism-corrected C-index of 0.72. Time-dependent AUCs were 0.77 (95% CI 0.70–0.83) at 12 months and 0.77 (95% CI 0.72–0.82) at 24 months (Figure 4). Calibration plots confirmed near-linear alignment up to approximately 50% predicted risk (Figure 5). Intercepts were −0.002 (95% CI −0.28 to 0.27) for 12 months and 0.03 (95% CI −0.20 to 0.25) at 24 months, suggesting no systematic miscalibration. Calibration slopes were 1.30 (95% CI 0.75–1.31) and 1.05 (95% CI 0.82–1.28), respectively. Brier scores (0.060 and 0.093) also indicated strong overall calibration accuracy. Predicted risks were more dispersed for FoVOx2 than in the temporal validation set, with a heavier right-tail and a larger share of patients >0.20 risk probability (Figure 6; Appendix Figure 4). At the 20% thresholds, sensitivity and specificity were 0.23 and 0.96 at 12 months and 0.46 and 0.86 at 24 months; corresponding metrics at the 5% threshold, as well as PPV and NPV, are provided in Supplementary Material (Appendix Table 3).

## Discussion

Using nationwide Swedish register data, this study tested a new external validation of FoVOx – a novel scalable tool for predicting violent reoffending after discharge from forensic psychiatric care – and developed an updated version using additional clinical factors. In total, 906 patients were followed up for a mean of 52 months with 172 (19%) violent reoffences. The following principal findings emerged.

First, FoVOx demonstrated moderate to good performance in external validation, based on discrimination and calibration, when applied to a contemporary cohort of sentenced forensic patients. There was some reduction in performance compared to the development sample (where AUCs were 0.77 at 12 and 24 months versus AUCs of 0.69 and 0.71, respectively, in this external validation), which was not unexpected in a newer cohort. The findings indicate that FoVOx maintains predictive utility despite changes in clinical practice and patient mix over time. Reasons for this include the choice of baseline predictors, which was based on theory and empirical testing, and methodological aspects of the original study (which avoided overfitting, for example, and was sufficiently statistically powered).

Second, model updating was shown to be potentially useful. FoVOx2 did not include variables considered as more difficult to assess reliably in routine practice, and added clinically relevant predictors, including clozapine treatment and two categories of personality disorder. These adjustments can additionally improve both the interpretability and feasibility of the tool. The inclusion of new treatment-related and diagnostic variables increased clinical relevance and better aligned the model with current forensic practice.

Third, incorporating these new predictors added incrementally to model performance. In the current sample, FoVOx2 achieved higher discrimination (AUCs 0.77) compared with the original model in the current validation (AUCs 0.69 and 0.71) and good overall calibration. Together, the findings illustrate the value of updating actuarial tools and provide a model for testing the incremental value of new risk markers, ensuring utility as population and clinical practice evolve.

Another finding was that the absence of previous psychiatric inpatient treatment was associated with lower risk of violent recidivism, which may reflect differences in underlying clinical trajectories within the forensic population. Individuals without prior inpatient care may include those whose offending occurred during an early or untreated phase of illness, with subsequent treatment leading to substantial risk reduction. This interpretation is supported by evidence that the risk of violence is elevated during first-episode or untreated psychosis and declines following initiation of treatment [48]. In contrast, offending despite previous treatment may indicate a more persistent or complex risk profile, such as treatment resistance, comorbid antisocial traits, or variable adherence. This aligns with typologies that separate out two groups: those with longstanding antisocial behaviour preceding illness onset and those whose violence emerges in the context of acute or untreated psychosis [49]. For those with comorbid antisocial traits, further compulsory treatment may yield smaller reductions in recidivism risk.

### Clinical implications

With AUCs of 0.77, FoVOx2 demonstrated predictive performance comparable to or better than other validated violence risk models [15]. The updated models incorporated a medication variable (clozapine treatment), linking treatment status to reduced violence risk. Integrating medication data adds clinical depth and aligns with evidence that effective pharmacotherapy, particularly clozapine, can reduce violent behaviour in individuals with psychotic disorders [50, 51] although the predictors were not causally tested. The updated instrument provides a transparent, data-driven, and reproducible alternative to structured professional judgement tools, which rely on subjective ratings and often show limited inter-rater reliability, and may involve substantial opportunity costs. The efficiency and feasibility of the new tool makes it highly applicable for routine clinical decision-making [23, 52].

A further consideration concerns communication of risk. In line with the TRIPOD+AI recommendations, prediction models are intended to provide individualised risk probabilities, alongside measures of discrimination and calibration, rather than relying primarily on categories [53]. Although categorisation into “low-,” “medium-,” and “high”-risk bands may offer pragmatic advantages, such groupings can obscure quantitative differences in risk, reduce precision, and compromise calibration. Moreover, because different professionals and lay audiences may interpret risk categories differently, these groupings risk losing practical meaning. Presenting predicted probabilities directly – as percentages – offers greater transparency and provides a common reference point for clinicians, courts, and decision-makers, helping to contextualise risk without imposing arbitrary thresholds. It is important to note that these estimates reflect the probability of a specified outcome and do not incorporate other dimensions of risk relevant to decision-making, such as severity or societal impact. In addition, violent outcomes are heterogeneous in impact and context, and a binary outcome does not capture differences in seriousness or consequences of reoffending. Predicted probabilities should therefore not be interpreted in isolation as decision thresholds, as clinical and legal decisions must also consider proportionality, imminence, clinical need, public interest, and the nature of potential harm. This approach supports the use of such probabilistic estimates to augment and supplement clinical judgement, rather than determining decision-making.

### Strengths and limitations

This study benefits from comprehensive national coverage, robust censoring methods, and large cohorts. Using offence dates rather than convictions minimised bias from judicial delays. Limitations include reliance on administrative data, potential diagnostic misclassification, and partial temporal overlap between development and validation cohorts. In addition, generalisability to other jurisdictions may be limited, particularly where discharge practices differ, as our definition of discharge reflects total discharge from forensic services rather than solely inpatient discharge. Although classification metrics such as PPV and NPV are reported, their interpretation depends on outcome prevalence, threshold choice and group sizes, and should therefore be interpreted with caution. Furthermore, the outcome of violent reconviction is heterogeneous and does not capture differences in severity or context of violence. Future external validations in independent and other national forensic samples are needed to confirm generalisability. Feasibility studies can examine whether and how FoVOx2 can complement decision-making and can consider how to link risk ratings with management. Future refinements may include recalibration in different jurisdictions (to account for different reoffending rates), evaluation of performance across subgroups, and exploration of whether incorporating additional clinical or contextual factors could further improve predictive accuracy and clinical utility.

## Conclusion

In this nationwide temporal validation, the original FoVOx model maintained moderate discriminatory accuracy and calibration, with modest overprediction at the upper end of predicted risk at 12 months and slight underprediction at 24 months. The updated model, FoVOx2, demonstrated improvements in model performance over the original version of the tool. Our findings therefore extend the FoVOx framework to a more contemporary cohort of forensic patients and support its use as an empirically grounded and clinically interpretable tool for discharge planning and violence risk management in forensic mental health. The generalisability of these findings to other settings will depend on differences in legal frameworks and clinical practice; however, the model may be adaptable to other contexts through recalibration to local outcome rates and population characteristics. Predicted probabilities should be interpreted within their specific decision-making context, where judgements may vary across clinical and legal decision-makers and be influenced by the perceived consequences of adverse outcomes. Finally, moving away from categorical labels toward reporting continuous probability estimates may further enhance interpretability and practical application, allowing risk estimates to augment clinical judgement rather than replacing it.

## Supporting information

10.1192/j.eurpsy.2026.12223.sm001 Sivak et al. supplementary material Sivak et al. supplementary material

## Data Availability

The data supporting the findings of this study are not publicly available because they contain sensitive personal and clinical information. Data sharing is restricted to protect participant confidentiality and to comply with ethical approvals and the General Data Protection Regulation.

## Supplementary material

The supplementary material for this article can be found at http://doi.org/10.1192/j.eurpsy.2026.12223.

## Use of AI tools

ChatGPT (version 5.1 and 5.2; OpenAI, https://chat.openai.com) was used exclusively for language editing and proofreading of the manuscript. No AI tool was used for study design, data collection, data analysis, interpretation of results, or generation of scientific content. The tool was accessed via the public web interface, without any modification, customization, or addition of proprietary or study-specific data to its training corpus. ChatGPT was used between 21 November and 21 December 2025. The authors critically reviewed and edited all AI-assisted text and take full responsibility for the content of the manuscript. No competing interests or biases are considered to arise from the use of this tool.

## [Table 1] Long description

Starting from the top row, the table presents three columns: No violent recidivism, Violent recidivism, and Overall. Number of observations is 649, 118, and 767 respectively. Time to relapse or censoring shows mean months (standard deviation): 47.9 (33.3) for no recidivism, 22.1 (20.6) for violent recidivism, and 43.9 (33.0) overall. Median months [minimum, maximum]: 41.5 [0.131, 119] for no recidivism, 16.0 [0.296, 93.4] for violent recidivism, 36.1 [0.131, 119] overall. Sex distribution: Male n is 502 (77.3 percent), 104 (88.1 percent), 606 (79.0 percent); Female n is 147 (22.7 percent), 14 (11.9 percent), 161 (21.0 percent). Age at discharge mean years (standard deviation): 43.5 (13.7), 36.2 (9.75), 42.4 (13.4). Median years [minimum, maximum]: 41.0 [20.0, 92.0], 33.5 [22.0, 65.0], 39.0 [20.0, 92.0]. Duration of forensic psychiatric treatment mean months (standard deviation): 60.3 (34.0), 57.4 (39.9), 59.9 (35.0). Median months [minimum, maximum]: 55.2 [3.88, 227], 46.0 [3.75, 213], 54.0 [3.75, 227]. Personality disorder at discharge: No n is 552 (85.1 percent), 89 (75.4 percent), 641 (83.6 percent); Yes n is 97 (14.9 percent), 29 (24.6 percent), 126 (16.4 percent). Previous violent crime: No n is 75 (11.6 percent), 4 (3.4 percent), 79 (10.3 percent); Yes n is 574 (88.4 percent), 113 (96.6 percent), 688 (89.7 percent). Previous serious violent crime: No is 477 (73.5 percent), 84 (71.2 percent), 561 (73.1 percent); Yes is 172 (26.5 percent), 34 (28.8 percent), 206 (26.9 percent). Primary diagnosis at discharge: Schizophrenia spectrum disorder n is 355 (54.7 percent), 61 (51.7 percent), 416 (54.2 percent); Bipolar disorder n is 48 (7.4 percent), 11 (9.3 percent), 59 (7.7 percent); Unipolar depression n is 12 (1.8 percent), 0 (0 percent), 12 (1.6 percent); Anxiety disorder n is 9 (1.4 percent), 0 (0 percent), 9 (1.2 percent); Other n is 225 (34.7 percent), 46 (39.0 percent), 271 (35.3 percent). Drug use disorder at discharge: No n is 449 (69.2 percent), 49 (41.5 percent), 498 (64.9 percent); Yes n is 200 (30.8 percent), 69 (58.5 percent), 269 (35.1 percent). Lifetime drug use disorder: No n is 279 (43.0 percent), 28 (23.7 percent), 307 (40.0 percent); Yes n is 350 (53.9 percent), 89 (75.4 percent), 439 (57.2 percent); Missing n is 20 (3.1 percent), 1 (0.8 percent), 21 (2.7 percent). Alcohol use disorder at discharge: No n is 552 (85.1 percent), 100 (84.7 percent), 652 (85.0 percent); Yes n is 97 (14.9 percent), 18 (15.3 percent), 115 (15.0 percent). Employment before admission: No n is 555 (85.5 percent), 106 (89.8 percent), 661 (86.2 percent); Yes n is 88 (13.6 percent), 10 (8.5 percent), 98 (12.8 percent); Missing n is 6 (0.9 percent), 2 (1.7 percent), 8 (1.0 percent). Number of previous inpatient episodes (five or more): No n is 370 (57.0 percent), 54 (45.8 percent), 424 (55.3 percent); Yes n is 279 (43.0 percent), 64 (54.2 percent), 343 (44.7 percent). Duration of forensic psychiatric treatment (12 months or more): No n is 26 (4.0 percent), 9 (7.6 percent), 35 (4.6 percent); Yes n is 623 (96.0 percent), 109 (92.4 percent), 732 (95.4 percent).

Navigate back to [Table 1].

## [Table 2] Long description

Starting from the top, the table lists three columns: No violent recidivism, Violent recidivism, and Overall. Number of observations is 734, 172, and 906 respectively. Time to relapse or censoring shows mean months of 55.4 (standard deviation 40.5) for no recidivism, 28.5 (26.7) for violent recidivism, and 51.6 (40.1) overall. Median months are 47.6 [minimum 0.131, maximum 173] for no recidivism, 20.3 [0.296, 132] for violent recidivism, and 39.8 [0.131, 173] overall. Sex distribution is 77.9 percent male and 22.1 percent female for no recidivism, 86.0 percent male and 14.0 percent female for violent recidivism, and 79.5 percent male and 20.5 percent female overall. Age at discharge shows mean years of 43.6 (standard deviation 13.7) for no recidivism, 37.4 (10.5) for violent recidivism, and 42.4 (13.4) overall. Median years are 41.0 [18.0, 92.0] for no recidivism, 35.0 [20.0, 68.0] for violent recidivism, and 40.0 [18.0, 92.0] overall. Duration of forensic psychiatric treatment shows mean months of 56.1 (34.1) for no recidivism, 54.9 (39.1) for violent recidivism, and 55.9 (35.1) overall. Median months are 50.4 [2.17, 227] for no recidivism, 45.5 [3.75, 213] for violent recidivism, and 48.9 [2.17, 227] overall. Previous violent crime is present in 88.3 percent of no recidivism, 95.9 percent of violent recidivism, and 89.7 percent overall. Previous serious violent crime is present in 26.3 percent of no recidivism, 25.0 percent of violent recidivism, and 26.0 percent overall. Schizophrenia spectrum disorder is present in 71.1 percent of no recidivism, 72.1 percent of violent recidivism, and 71.3 percent overall. Affective disorder is present in 18.1 percent of no recidivism, 15.7 percent of violent recidivism, and 17.7 percent overall. Developmental disorder (I D or A S D) is present in 42.1 percent of no recidivism, 54.7 percent of violent recidivism, and 44.5 percent overall. A D H D is present in 11.2 percent of no recidivism, 16.9 percent of violent recidivism, and 12.3 percent overall. Antisocial personality disorder is present in 4.5 percent of no recidivism, 15.7 percent of violent recidivism, and 6.6 percent overall. Other personality disorders are present in 14.3 percent of no recidivism, 20.3 percent of violent recidivism, and 15.5 percent overall. Drug use disorder is present in 29.0 percent of no recidivism, 55.2 percent of violent recidivism, and 34.0 percent overall. Alcohol use disorder is present in 14.6 percent of no recidivism, 15.7 percent of violent recidivism, and 14.8 percent overall. Previous psychiatric inpatient treatment is present in 86.9 percent of no recidivism, 93.6 percent of violent recidivism, and 88.2 percent overall. Long-acting injectables are used in 34.1 percent of no recidivism, 29.1 percent of violent recidivism, and 33.1 percent overall. Oral antipsychotics are used in 29.0 percent of no recidivism, 29.1 percent of violent recidivism, and 29.0 percent overall. Clozapine is used in 12.1 percent of no recidivism, 3.5 percent of violent recidivism, and 10.5 percent overall. Mood stabilizers are used in 16.6 percent of no recidivism, 13.4 percent of violent recidivism, and 16.0 percent overall. Antidepressants are used in 29.8 percent of no recidivism, 19.8 percent of violent recidivism, and 27.9 percent overall. Each variable is presented with counts and percentages for each group.

Navigate back to [Table 2].

## [Figure 1] Long description

The left panel shows a density plot and histogram of linear predictor values for all individuals at 24 months. The x axis is labeled Linear Predictor Value, ranging from about minus three to two. The y axis is labeled Density, ranging from zero to about zero point four five. The distribution is unimodal and roughly symmetric, centered near minus zero point six. Overlaid text reports mean equals minus zero point six three, median equals minus zero point five seven, S D equals zero point eight eight, I Q R equals minus one point two one to zero point zero five. The right panel displays two overlaid density plots and histograms by violent recidivism status. The blue distribution represents no violent recidivism, with mean equals minus zero point seven two, median equals minus zero point six eight, S D equals zero point eight nine, I Q R equals minus one point three three to minus zero point zero two. The gold distribution represents violent recidivism, with mean equals minus zero point zero four, median equals zero point zero five, S D equals zero point six nine, I Q R equals minus zero point five one to zero point three six. The gold distribution is shifted rightward compared to blue, indicating higher linear predictor values for those with violent recidivism. The legend at the bottom right identifies blue as no violent recidivism and gold as violent recidivism.

Navigate back to [Figure 1].

## [Figure 2] Long description

The chart displays predicted risk categories on the x axis, ranging from 0-0.25 percent on the far left to greater than 50 percent on the far right, with intervals increasing by 2.5 percent up to 20 percent, then by 5 percent. The y axis shows percent of sample from 0 to 20. Bars are colored green for low risk (0-5 percent), yellow for medium risk (5-20 percent), and red for high risk (greater than 20 percent). The tallest bar is in the 2.5-5 percent category, reaching just above 20 percent of the sample. Three vertical dashed lines divide the chart into low, medium, and high risk groups. Above each group, text indicates observed offending rates: 4.5 percent offend for low risk, 16.3 percent offend for medium risk, and 31.6 percent offend for high risk. The distribution shows most of the sample falls into low and medium risk categories, with very few in the highest risk bins.

Navigate back to [Figure 2].

## [Figure 3] Long description

The left panel is titled Calibration at 12 months. The x axis is labeled Estimated probability, ranging from 0 to 0.2. The y axis is labeled Observed probability, ranging from 0 to 0.3. Purple dots with vertical error bars represent observed data points. A solid purple line shows the calibration curve, with a shaded region indicating the confidence interval. A dashed black line represents the ideal calibration where observed equals estimated probability. The calibration curve generally follows the diagonal but deviates upward at higher probabilities. The right panel is titled Calibration at 24 months. The x axis is labeled Estimated probability, ranging from 0 to 0.4. The y axis is labeled Observed probability, ranging from 0 to 0.6. The panel contains purple dots with error bars, a solid purple calibration curve, and a shaded confidence interval. The dashed black line again marks the ideal calibration. The calibration curve closely follows the diagonal at lower probabilities but rises above it at higher probabilities, with wider confidence intervals.

Navigate back to [Figure 3].

## [Table 3] Long description

Beginning at the top row, the predictors are listed in the first column: Sex (female), Age at discharge (per 1-year older), Previous violent crime, Schizophrenia spectrum disorder, Alcohol use disorder, Substance use disorder other than alcohol, Antisocial personality disorder, Personality disorder other than antisocial, Clozapine treatment, and Absence of previous psychiatric inpatient care. The second column displays hazard ratios: 0.61, 0.97, 3.90, 1.19, 1.25, 2.34, 3.37, 1.70, 0.28, and 0.58 respectively. The third column shows 95 percent confidence intervals: 0.39–0.95, 0.95–0.98, 1.82–8.35, 0.83–1.69, 0.82–1.91, 1.70–3.22, 2.18–5.23, 1.13–2.54, 0.12–0.63, and 0.31–1.07. The fourth column contains p-values: 0.029, less than 0.001, less than 0.001, 0.348, 0.309, less than 0.001, less than 0.001, 0.011, 0.002, and 0.082. Significant predictors with hazard ratios above 1 and p-values less than 0.05 include previous violent crime, substance use disorder other than alcohol, antisocial personality disorder, and personality disorder other than antisocial. Protective factors with hazard ratios below 1 and significant p-values include sex (female), age at discharge, and clozapine treatment.

Navigate back to [Table 3].

## [Figure 4] Long description

The left panel is titled ROC Curve 12 Months. The x-axis is labeled 1 minus Specificity from 0.00 to 1.00. The y-axis is labeled Sensitivity from 0.00 to 1.00. A blue ROC curve rises steeply then gradually, staying above the diagonal reference line. Dotted horizontal and vertical lines mark 5 percent and 20 percent cut-off points. The A U C value 0.766 is shown in large blue text. The right panel is titled ROC Curve 24 Months. Axes are identical to the left panel. The blue ROC curve again rises above the diagonal reference line, with similar cut-off markers. The A U C value 0.771 is displayed in blue. Both panels show the ROC curve consistently outperforming the reference line, indicating good model discrimination.

Navigate back to [Figure 4].

## [Figure 5] Long description

The left panel is titled Calibration at 12 months. The x-axis is labeled Estimated probability, ranging from 0.0 to 0.5. The y-axis is labeled Observed probability, ranging from 0.0 to 0.6. A dashed diagonal line represents perfect calibration. Purple points with vertical error bars show observed data. A solid purple line fits the data, with a shaded region indicating confidence intervals. The line closely follows the diagonal at lower probabilities but deviates upward at higher probabilities. The right panel is titled Calibration at 24 months. The x-axis is labeled Estimated probability, ranging from 0.0 to 0.75. The y-axis is labeled Observed probability, ranging from 0.0 to 1.0. The dashed diagonal line again represents perfect calibration. Purple points with error bars and a solid purple fit line with shaded confidence intervals are shown. The fit line tracks the diagonal more closely across the range, with slight upward deviation at higher probabilities. Both panels illustrate the relationship between estimated and observed probabilities for FoVOx2 at different time points.

Navigate back to [Figure 5].

## [Figure 6] Long description

The top panel, labeled FoVOx2 updated, displays a histogram with the x-axis labeled Predicted 24-month risk ranging from 0.0 to 0.6 and the y-axis labeled Count ranging from 0 to 90. The majority of bars are concentrated between 0.0 and 0.15, peaking at just above 90 counts, then gradually decreasing with a long right tail extending to 0.6. The bottom panel, labeled Temporal validation, shows a similar histogram with the same axes. Most bars are also concentrated between 0.0 and 0.15, peaking at just above 90 counts, then decreasing more sharply than the top panel, with very few counts above 0.3. Both panels illustrate that most predicted risks are low, but the FoVOx2 updated panel has a longer tail of higher risk predictions compared to the temporal validation panel.

Navigate back to [Figure 6].
